# Delay in the dispersal of flocks moving in unbounded space using long-range interactions

**DOI:** 10.1038/s41598-018-34208-x

**Published:** 2018-10-26

**Authors:** Martín Zumaya, Hernán Larralde, Maximino Aldana

**Affiliations:** 1 Instituto de Ciencias Físicas, Universidad Nacional Autónoma de México, Avenida Universidad s/n, Colonia Chamilpa, Código Postal, 62210 Cuernavaca, Morelos Mexico; 2 Centro de Ciencias de la Complejidad, Universidad Nacional Autónoma de México, Ciudad de México, Mexico

## Abstract

Since the pioneering work by Vicsek and his collaborators on the motion of self-propelled particles, most of the subsequent studies have focused on the onset of ordered states through a phase transition driven by particle density and noise. Usually, the particles in these systems are placed within periodic boundary conditions and interact via short-range velocity alignment forces. However, when the periodic boundaries are eliminated, letting the particles move in open space, the system is not able to organize into a coherently moving group since even small amounts of noise cause the flock to break apart. While the phase transition has been thoroughly studied, the conditions to keep the flock cohesive in open space are still poorly understood. Here we extend the Vicsek model of collective motion by introducing long-range alignment interactions between the particles. We show that just a small number of these interactions is enough for the system to build up long lasting ordered states of collective motion in open space and in the presence of noise. This finding was verified for other models in addition to the Vicsek one, suggesting its generality and revealing the importance that long-range interactions can have for the cohesion of the flock.

## Introduction

Collective motion is one of the most spectacular displays of coordinated behavior in nature, exhibited by systems of very different kinds, ranging from cell populations to various species of insects and vertebrates, such as flocks of starlings, sheep herds, fish shoals and human crowds^1,2^. The coordinated motion of the system as a whole, where all individuals form a localized group in space and move approximately in the same direction, is an emergent property resulting from the interactions between all the individuals (or “particles”) comprising the system. While the long-term goal of the collective motion of many species of animals is to forage or migrate, this is not always the case. For example, marching locusts, when placed within a 2D circular track start moving in a collective way at high enough density, without any apparent long-term goal such as migration or foraging^3^. Starling flocks and fish schools also display collective motion which is not necessarily consistent with migration, foraging or looking for a suitable habitat^4–6^. As far as we know, there is no consensus among biologist about the evolutionary reasons for the collective motion of many species, although for some other species the reasons are as diverse as cannibalism for cannibal locust^7^, foraging for monkeys^8,9^ or migration for ducks and monarch butterflies. However, even in the clear cases in which the reason is migration, this activity can last days or weeks, and in the meantime the group has to remain together. To the best of our understanding, most of the flocking models that have been proposed describe the mechanisms that generate spontaneous coordinated motion during relatively short periods of time without considering the ultimate long-term goal of such collective motion.

**Figure 1:**
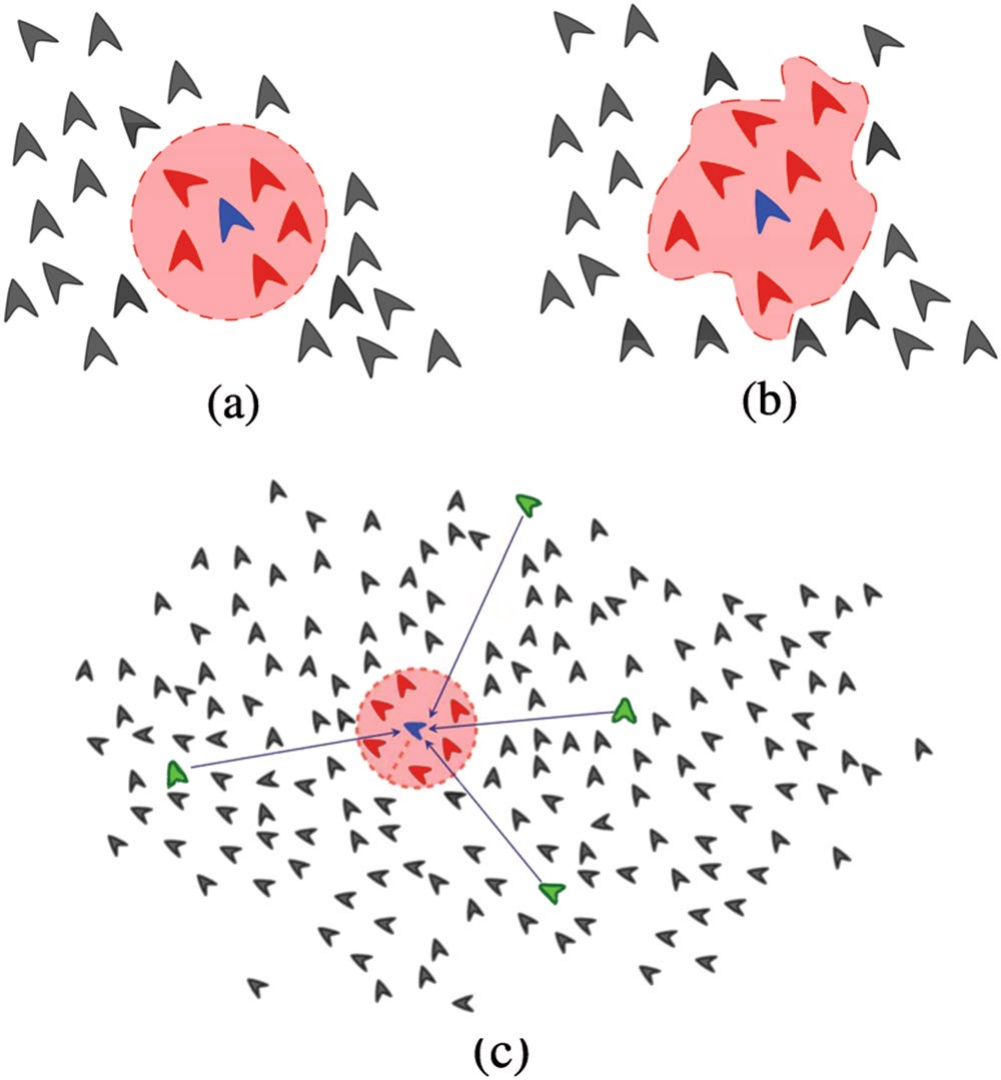
Schematic representation of different interactions for an arbitrary particle *p*_*i*_ (represented in blue). Short-range interactions can be defined either (**a**) metrically, where each particle interacts with the ones falling within a circular neighborhood of fixed radius *r*_0_, or (**b**) topologically where each particle interacts with its first *α*_*l*_ neighbors regardless of their relative distance. (In this example *α*_*l*_ = 7). In each case, the red particles constitute the local neighborhood *U*_*i*_(*t*) of *p*_*i*_, whereas the gray particles outside the shaded area constitute the complementary set ${U}_{i}^{\ast }(t)$. (**c**) In addition to short-range interactions within the local neighborhood *U*_*i*_(*t*), each particle *p*_*i*_ also has a certain number *κ*_*i*_ of long-range interactions with distant neighbors, indicated here by the green particles which constitute the distant neighborhood *L*_*i*_(*t*) of *p*_*i*_. (In this example *κ*_*i*_ = 4).

In the last decades, various models of collective motion have been proposed and a number of studies have been carried out, both theoretical^10–17^ and empirical^4,18–21^. In these, the main problem has been to determine the nature of the interactions between particles and the mechanisms needed for the system to build up states of collective motion. Most of these models are based on *local* (or short-range) alignment interactions where individuals modify their direction of motion to match the average direction of their immediate neighbors, plus some noise^11,22,23^. These short-range interactions can be defined either metrically, where each particle interacts with all others within some distance *r*_0_ (like in the standard Vicsek model, see Fig. 1(a)), or topologically where particles interact with a fixed number *α*_*l*_ of their first neighbors regardless of their relative distance (like in Ballerini *et al*., see Fig. 1(b)). There exists empirical evidence supporting both metric and topologic interactions^3,4,7,24–26^, and which kind is present strongly depends on the nature of the particles the system is made of^1^. Other models also introduce repulsion and attraction between particles in order to avoid collisions and prevent the system from breaking apart^16,27–30^.

Although models based on local interactions between neighboring particles are able to reproduce important features of collective motion, most of them are defined within periodic or reflective boundary conditions^22^, or consider the system starting from an already ordered stationary state^11^. However, in none of these models the system is able to maintain order or cohesion in open space, (i.e. when the boundaries are removed), unless explicit attraction terms very strong or of infinite range are introduced^27–31^. One of the first models introducing long-range interactions between particles was proposed by Couzin *et al*.^27^. This model, which reproduces highly realistic motions, incorporates repulsion, orientation and attraction zones for each particle (see Supplementary Note I and Supplementary Fig. S1). Nevertheless, the range of the orientation and attraction zones in Couzin’s model must be comparable to, or even larger than, the size of the entire system in order to reach ordered states in open space (see Supplementary Fig. S2). This implies that every particle has to interact with all the other ones in the system through infinite-range attractive forces spanning the entire group to prevent the system from breaking apart in open space. Another recent model that implements long-range interactions between particles was proposed by Pearce *et al*.^12^. In this model, each particle represents a bird which perceives around it bright and opaque areas of sky, being the latter regions the ones occupied by other birds in the flock. Each bird, in addition to interacting with its first neighbors, tends to move essentially in the direction of the most opaque regions of sky because it is in those regions where the majority of the other birds are. As recognized by Pearce and his coauthors, this bias towards the opaque regions effectively introduces long-range interactions of infinite range between almost all the birds in the flock. One of the advantages of this model is that the flock never breaks apart when moving in open space (i.e. without periodic boundaries).

Another strategy that has been proposed to prevent the flock from breaking apart is to provide the particles with different rules of motion depending on whether they are within the flock (in the bulk) or on its border (the region that separates the bulk of the flock from infinite empty space)^32–34^. In these models, particles within the flock interact only with their first neighbors, whereas particles on the border, in addition to the first-neighbor interactions, also have a bias to move either towards the interior of the flock or towards the center of mass of other nearby particles on the border. In this way, particles on the flock’s border exert a sort of surface tension that prevents the flock from fracturing. This is reminiscent of the way in which bacterial colonies move and expand. As it has been shown that bacterial swarms move by chemically regulating the surface tension of the boundary layer that separates the swarm from the surrounding medium^35,36^. The border-bias strategy mentioned above has actually been implemented in robot swarms^37^. Both numerical simulations and robot swarms show that, unless the bias of the bordering particles is very strong, the group eventually breaks apart.

The important point to stress here is that flocking models based only on local interactions between the particles have not been able to sustain cohesion of the flock for long periods of time, as has been already recognized by several authors^12,32,38^. This problem is often circumvented by implementing either periodic boundary conditions or infinite-range attractive interactions between the particles. However, these long-range interactions are not always explicitly implemented, rather, they are implicit in the effects of opacity, the implementation of border biases or in other complicated cohesion inducing mechanisms. The main purpose of this work is to explicitly investigate the role that long-range alignment interactions play on the cohesiveness of the flock. For this, we propose a variation of the Vicsek model^22^ and the Inertial Spin model^11^ where the particles are free to move in open space and in which, in addition to the usual local alignment interactions (which can be either metric or topological), a small fraction of long-range alignment interactions are explicitly introduced. Previous work has shown that introducing long-range interactions between static spins with continuous degrees of freedom and fixed to a lattice (specifically the XY-model and other 2D spin models with Vicsek-like alignment rules), generates equilibrium order-disorder phase transitions^39–43^. Furthermore, it has been shown the Vicsek model with certain local topological interactions (based on Voronoi tessellations) is able to build up ordered states, although the particles eventually spread out reaching states of zero-density^13,29,44^. However, here we are not discussing the effect that long-range interactions have on the existence or absence of noise-driven phase transitions. Rather, we are interested in the conditions that prevent this non-equilibrium system of moving particles from dispersing or breaking apart in the absence of boundaries.

The rationale for introducing long-range interactions in the flock is the following. Imagine walking with a friend in the middle of a crowd, with people all around you, pushing you both back and forth. Due to this pushing and pulling, you and your friend eventually get separated while the crowd keeps advancing in some direction. Clearly, you will be interacting with the people immediately around you (the ones pushing you through local forces), but you will also be interacting with your friend even when you are already separated because you will try to follow her across the crowd. These are the long-range interactions which in our model are implemented through a random interaction network connecting different particles regardless of their separation. For a real flock, we do not expect individuals to follow their specific “friends”. This is just a metaphor that captures the essence of another quite reasonable assumption: in a flock, each bird, in addition to interacting with its first neighbors (either metrically or topologically), is also able to sense different parts of the entire flock, whether distant or not, and move accordingly. It would be naïve to think that each bird can only see the seven o so birds in its immediate vicinity^4^ and not be aware of the motion of different parts of the flock. It is this sensing of the motion of the group that we try to capture with the random long-range interaction network. This is somewhat analogous to sensing the bright and opaque regions of the sky, and being biased essentially in the direction of the more opaque regions^12^; or having particles on the flock’s border identify other particles on the border and being biased towards their center of mass. The difference with previous work is that in our model we explicitly implement long-range interactions between particles and measure the cohesion of the flock as a function of the number of long-range interactions. One of the important results of this work is that not every bird has to be aware of the motion of the flock to prevent its fracture. It is enough that just a few birds pay attention to the motion of some parts of the flock (not even the entire flock) to prevent it from quickly disintegrating.

## Model Definition

The model consists of a set *F*–the flock–of *N* self-propelled particles, *F* = {*p*_1_, *p*_2_, …, *p*_*N*_} moving in open space, either in two or three dimensions. By “open space” we mean that the motion of the particles is not restricted by any boundary. Each particle *p*_*n*_ is characterized by its position **r**_*n*_, velocity **v**_*n*_, and speed $\Vert {{\bf{v}}}_{{\bf{n}}}(t)\Vert ={v}_{0}$ which is the same at all times so that only the particle’s direction of motion is modified by interactions with other particles in the system.

### Short and Long-range Interactions

Let *U*_*i*_(*t*) be the set of all particles that locally interact with particle *p*_*i*_ at time *t*. For the case of metric interactions, *U*_*i*_(*t*) consists of all particles within a vicinity of radius *r*_0_ around *p*_*i*_ (Fig. 1(a)), whereas for topological interactions *U*_*i*_(*t*) consists of the first *α*_*l*_ = 7 nearest neighbors of *p*_*i*_ (Fig. 1(b)). We choose *α*_*l*_ = 7 because this is the average number of first-neighbor interactions reported for starlings^4,24^. Although this number may change for different bird species, it is also more or less the number of first-neighbor interactions as computed through a Voronoi tessellation^13,32,33,44^. The difference between metric and topological interactions is relevant under expansions and contractions of the group. Clearly, for the metric case the number of particles within a vicinity of *fixed radius r*_0_ changes as the system expands or contracts, whereas for the topological case the number *α*_*l*_ of first nearest neighbors remains the same regardless of the actual size of the group. In either case, *U*_*i*_(*t*) contains all the particles that interact with particle *p*_*i*_ at time *t* only through local (or short-range) interactions. Therefore, we will refer to *U*_*i*_(*t*) as the *local neighborhood* of particle *p*_*i*_. All the particles outside the local neighborhood *U*_*i*_(*t*) form the complementary set ${U}_{i}^{\ast }(t)$, such that ${U}_{i}(t)\cap {U}_{i}^{\ast }(t)=\varnothing$ and ${U}_{i}(t)\cup {U}_{i}^{\ast }(t)=F$. The particles in the set ${U}_{i}^{\ast }(t)$ are the potential long-range neighbors of *p*_*i*_ (Fig. 1(c)). In order to define the long-range interactions for this particle we randomly choose a set of *κ*_*i*_ particles in the complementary set ${U}_{i}^{\ast }(t)$, where *κ*_*i*_ is in turn a random number drawn from a Poisson distribution with average *κ*. The parameter *κ* then determines the average number of long-range interactions per particle. Let us denote as ${L}_{i}(t)\subset {U}_{i}^{\ast }(t)$ the set of these *κ*_*i*_ long-range neighbors that will interact with *p*_*i*_. We will refer to *L*_*i*_(*t*) as the *distant neighborhood* of particle *p*_*i*_, and to *κ* as the *average long-range connectivity*. Note that for *κ* = 0 only local interactions exist in the system (the ones defined in the sets *U*_*i*_(*t*) for each particle).

Note that the number *κ*_*i*_ of distant neighbors that interact with each particle *p*_*i*_ changes from one particle to another (*κ*_*i*_ is randomly chosen from a Poisson distribution and assigned to *p*_*i*_). Therefore, it is not necessary in our model that all particles have the same sensory capabilities. However, our numerical simulations produce nearly the same results if we choose *κ*_*i*_ = *κ* for all particles. Additionally, the particles in the distant neighborhood *L*_*i*_(*t*) of *p*_*i*_ can be the same throughout time (quenched dynamics) or they can change at every time step (annealed dynamics). The quenched case would correspond to a situation in which each particle *p*_*i*_ is following a specific set of “friends” (as in the metaphor above, where the person in the crowd is following his particular friend). In contrast, the annealed case would correspond to each particle *p*_*i*_ following different parts of the flock at different times. In either case, choosing a set *L*_*i*_(*t*) of long-range neighbors for each particle *p*_*i*_ in the system defines an Erdös-Rényi interaction network, characterized by a Poisson degree distribution with average *κ*^45^. In the quenched case the long-range network is fixed throughout time, whereas in the annealed case this network is rewired at each time step. Here we present results for the annealed case. This choice is arbitrary, as we are not aware of any empirical result indicating how long-range interactions, if any, are distributed among particles in a flock. However, we consider that the annealed case may be a better representation of the way in which a given particle senses distant parts of the flock.

### System dynamics

Once the local neighborhood *U*_*i*_(*t*) and the distant neighborhood *L*_*i*_(*t*) have been defined for each particle *p*_*i*_ in the flock at time *t*, we define the short-range signal **V**_*i*_(*t*) and the long-range signal ϒ_*i*_(*t*) that particle *p*_*i*_ receives at time *t* as

1a $${{\bf{V}}}_{i}(t)=\frac{1}{{k}_{i}(t)}\sum _{{p}_{j}\in {U}_{i}}\,{{\bf{v}}}_{j}(t),$$

1b $${\Upsilon }_{i}(t)=\frac{1}{{\kappa }_{i}}\sum _{{p}_{j}\in {L}_{i}}{{\bf{v}}}_{j}(t),$$

where *k*_*i*_ and *κ*_*i*_ are the cardinalities of *U*_*i*_(*t*) and *L*_*i*_(*t*), respectively. The evolution of the system is given by the simultaneous update of the directions and positions of all the particles in the system according to the rules

2a $${{\bf{s}}}_{i}(t)=\omega {{\bf{V}}}_{i}(t)+\mathrm{(1}-\omega ){\Upsilon }_{i}(t),$$

2b $${{\bf{x}}}_{i}(t)=\frac{{{\bf{v}}}_{i}(t)\times {{\bf{s}}}_{i}(t)}{\Vert {{\bf{v}}}_{i}(t)\times {{\bf{s}}}_{i}(t)\Vert },$$

2c $${\theta }_{i}(t)={\cos }^{-1}(\frac{{{\bf{v}}}_{i}(t)\cdot {{\bf{s}}}_{i}(t)}{{v}_{0}\Vert {{\bf{s}}}_{i}(t)\Vert })+\eta \,{\xi }_{i}(t),$$

2d $${{\bf{v}}}_{i}(t+{\rm{\Delta }}t)= {\mathcal R} \{{{\bf{x}}}_{i}(t);{\theta }_{i}(t)\}({{\bf{v}}}_{i}(t))$$

2e $${{\bf{r}}}_{i}(t+{\rm{\Delta }}t)={{\bf{r}}}_{i}(t)+{{\bf{v}}}_{i}(t+{\rm{\Delta }}t){\rm{\Delta }}t,$$

where the operator ${\mathcal R} \{{\bf{x}};\,\theta \}({\bf{v}})$ represents the rotation of a vector **v**, around an axis **x** by an angle *θ* in space, described with the quaternions formalism^46^. Eq. (2a) defines the total signal **s**_*i*_(*t*) that the particle receives from its local and distant neighborhoods. In this equation, the parameter *ω* ∈ [0, 1] represents the relative weight between short-range and long-range interactions, and allows the system to pass from purely short-range interactions when *ω* = 1, to only long-range interactions when *ω* = 0. All the results presented here are for *ω* = 1/2. Eq. (2b) defines the instantaneous axis of rotation **x**_*i*_(*t*) around which the velocity **v**_*i*_(*t*) of the particle will be rotated, whereas Eq. (2c) gives the amount *θ*_*i*_(*t*) of this rotation. (In 2D the vector **x**_*i*_(*t*) is always perpendicular to the plane of motion of the system.) The parameter *ξ*_*i*_(*t*) appearing on the right-hand side of Eq. (2c) is a random variable uniformly distributed in the interval [−*π*, *π*] and represents the noise in the perception of the particle. This noise has an intensity *η*, which is a parameter taking a constant value in the interval *η* ∈ [0, 1]. Eq. (2d) gives the new velocity of the particle at time *t* + Δ*t* obtained by rotating the velocity at the previous time step, **v**_*i*_(*t*), by an angle *θ*_*i*_(*t*) around the axis **x**_*i*_(*t*). Finally, Eq. (2e) gives the new position of the particle at time *t* + Δ*t*. It is important to note that for *κ* = 0 this model transforms into the standard Vicsek model (except for the periodic boundary conditions). In the Supplementary Note II we summarize the values of all the parameters used in the model. In what follows we present results for the 3D-case, but a completely analogous behavior is obtained in 2D (see the Supplementary Note IV).

## Results

**Figure 2:**
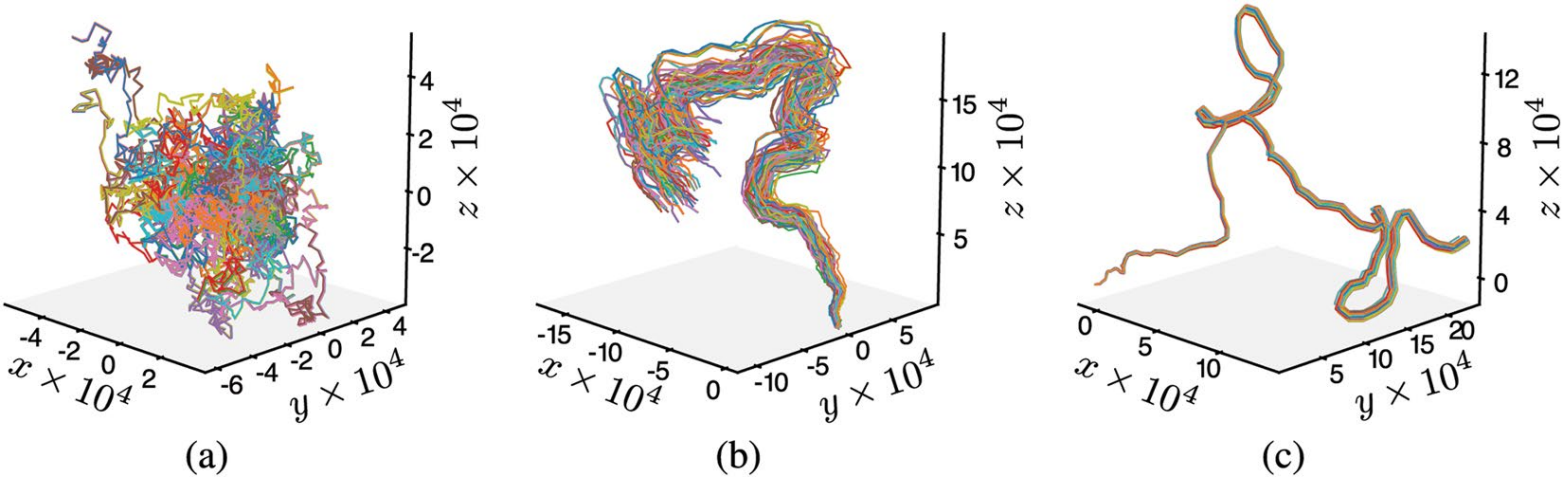
Typical trajectories of systems moving in open space with *N* = 512 particles, topological short-range interactions, fixed noise intensity *η* = 0.15, initial density *ρ*_0_ = 0.3 and different values of *κ*. (**a**) For *κ* = 0 the system is not able to organize into an ordered state even when *ρ*_0_ and *η* take values corresponding to the ordered phase of the standard Vicsek Model with periodic boundary conditions. (**b**) For *κ* = 0.01 the system becomes ordered and forms a spatially localized group. Note the noise-driven turns performed by the entire group. (**c**) For *κ* = 0.1 the system remains more compact and ordered for longer periods of time. All the trajectories were computed for 10^6^ time steps.

**Figure 3:**
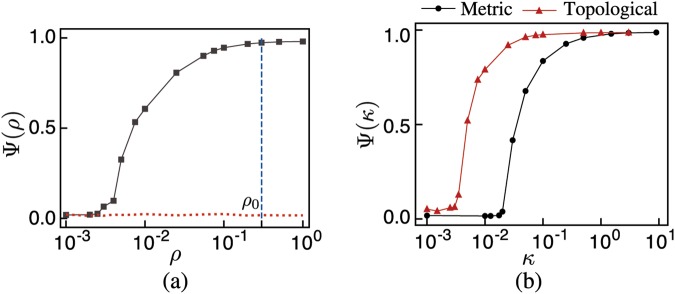
(**a**) Order parameter Ψ as a function of the density *ρ* for the 3D Vicsek model with periodic boundary conditions (black curve) and in open space (red dotted line). Note the complete absence of ordered states for the case without boundaries. *ρ*_0_ = 0.3 is the initial density used to generate the plots in Fig. 2. This value corresponds to highly ordered states in the standard Vicsek model. (**b**) Ψ as a function of the average long-range connectivity *κ* for systems moving in open space with short-range interactions implemented metrically (black curve with circles) and topologically (red curve with triangles). In both cases, note the onset of collective order for relatively low values of *κ*. The complete set of the parameters used in the simulations is listed in Supplementary Tables S2 and S3.

Figure 2 shows typical trajectories for systems moving in open space with *N* = 512 particles, topological short-range interactions and different values of the average long-range connectivity *κ*. In the three cases shown in this figure the system starts with the particles oriented in random directions and placed inside a box of sides ${L}_{0}=\sqrt[3]{N/{\rho }_{0}}$, being *ρ*_0_ the initial density. This density was chosen in such a way that the system would have reached a high degree of order in the standard Vicsek model with periodic boundary conditions (see Fig. 3(a)). Figure 2(a) shows the effect of removing the periodic boundary conditions in the standard Vicsek model. It is apparent that the system breaks apart, with each particle performing a random walk independently from the other ones. However, for non-zero values of *κ* even as small as *κ* = 0.01 (only one particle out of 100 has a long-range connection), the system is able to organize and move coherently for a long period of time (Fig. 2(b)). Increasing the value of *κ* makes the system remain ordered and more compact for even longer times (Fig. 2(c)).

To quantify these observations we measure the amount of order in the system through the parameter Ψ defined as

3 $${\rm{\Psi }}=\mathop{{\rm{l}}{\rm{i}}{\rm{m}}}\limits_{t\to T}\,\frac{1}{N{v}_{o}}\langle |\sum _{i=1}^{N}{{\bf{v}}}_{i}(t)|{\rangle }_{\kappa },$$

where *T* is a long enough time to allow the system reach a steady state, whereas 〈⋅〉_*κ*_ represents the ensemble average over different realizations of the long-range interaction network. This parameter Ψ is commonly used to characterize the order-disorder transition in collective motion models^1,10,22,30^. Thus, Ψ ≈ 1 corresponds to an ordered system where all the particles move in approximately the same direction, whereas Ψ ≈ 0 corresponds to a disordered system where the particles move in random uncorrelated directions. It is important to distinguish between order and cohesion in a group of particles, for there can be order without cohesion and vice versa. For example, it has been shown that the Vicsek model with topological interactions (determined by Voronoi tessellations) reaches highly ordered states with Ψ ≈ 1, although the particles asymptotically spread out throughout time reaching a zero-density state^13,29,44^. By contrast, in the Couzin model with no repulsion, large attraction zone and high noise the particles remain cohesive but disordered, as they randomly move towards the center of the flock and get stuck. The order parameter defined in Eq. (3) measures the amount of order in the group, but has nothing to say about its cohesion. For that, other parameters measuring the spatial extent of the flock will be introduced below.

Figure 3(a) shows Ψ as a function of the particle density *ρ* for the standard Vicsek model with periodic boundary contitions (black curve) and in open space (red dotted line). Both curves were computed for systems with the same number of particles *N* = 4096 and noise intensity *η* = 0.15. The black curve shows the familiar phase transition in the standard Vicsek model with periodic boundary conditions. However, when the periodic boundary is removed and the particles are free to move in open space, the same system cannot reach ordered states for any value of the density *ρ* (red dotted line). The situation drastically changes when long-range interactions between the particles are introduced. Figure 3(b) shows Ψ as a function of the average long-range connectivity *κ* for systems moving in open space in two cases: when the short-range interactions are implemented metrically (like in the Vicsek model) and when they are implemented topologically (like in the Ballerini *et al*. model). In both cases the system is able to organize into ordered states for sufficiently large values of *κ* for the duration of the simulations. Actually, it appears from Fig. 3(b) that these ordered states emerge through a continuous phase transition driven by the average long-range connectivity *κ*. Interestingly, the values of *κ* for which ordered states appear are relatively low, going for the topological case as low as only one long-range connection per 100 particles.

**Figure 4:**
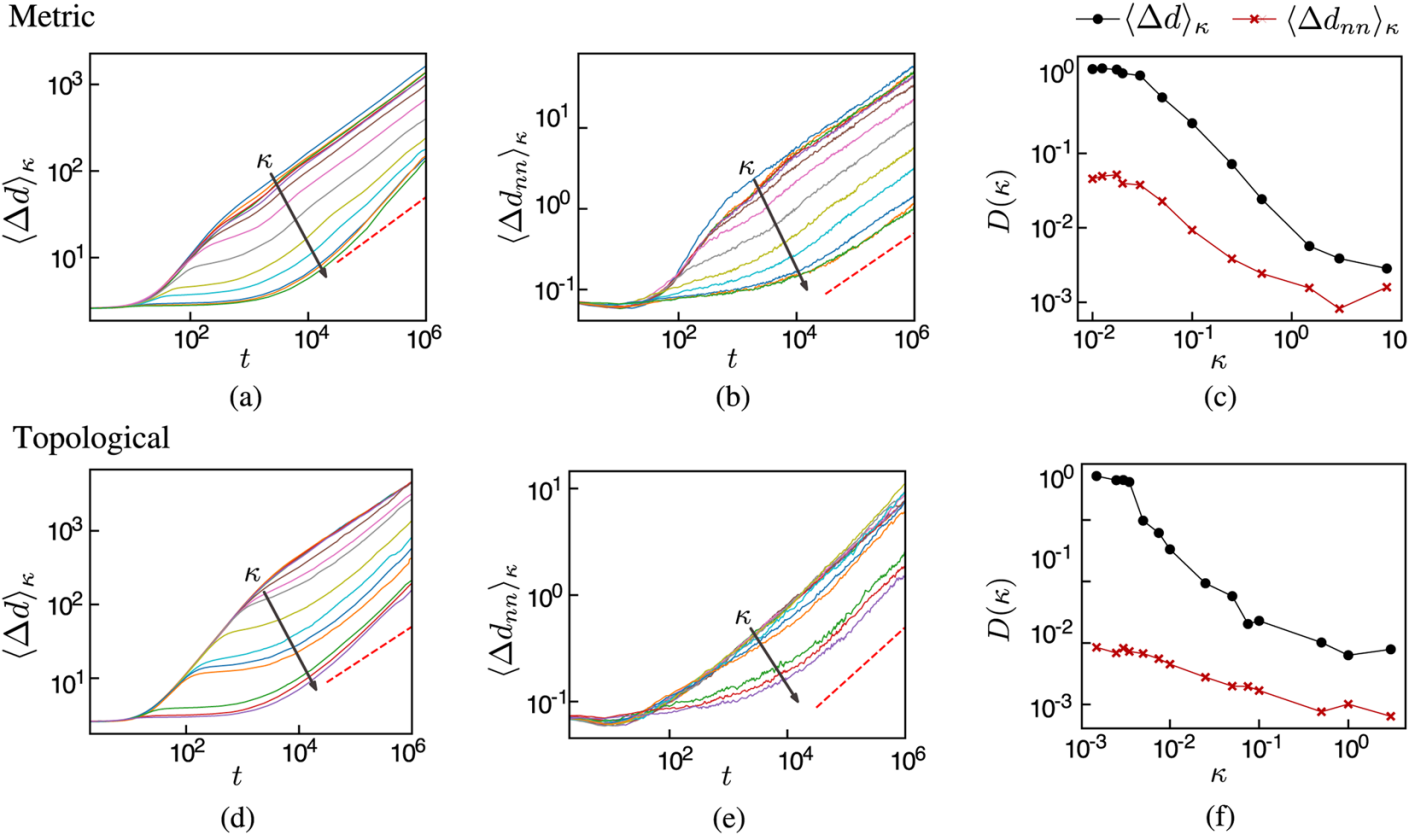
Average distance between particles 〈Δ*d*〉_*κ*_ and to the nearest neighbor 〈Δ*d*_*nn*_〉_*κ*_ as functions of time for different values of the average long-range connectivity *κ*. The short-range interactions were implemented metrically for the top panels, and topologically for the bottom panels. The arrows indicate the direction of increasing *κ* in the interval [0.0, 9.0] for metric short-range interactions and in the interval [0.0, 1.0] for topological ones. Note that these average distances asymptotically behave as *t*^*α*^ (the red dashed line corresponds to *α* = 1/2), indicating diffusive behavior. The two panels at the right show the diffusion coefficient as a function of *κ*. As *κ* increases, the diffusion coefficient decreases several orders of magnitude, showing the effect of the long-range connections on the expansion of the system. The simulations were carried out for systems with *N* = 4096, *η* = 0.15 and initial density *ρ*_0_ = 0.3.

It is important to mention that the flock moving in open space with long-range connections also displays a continuous order-disorder phase transition driven by the noise intensity *η* (see Supplementary Fig. S3 and the corresponding discussion in the Supplementary Note III). However, as the main objective of this work is to characterize de effect that long-range interactions have on the fracture of the flock, the noise intensity *η* was kept fixed in all our simulations at the value *η* = 0.15, which corresponds to the ordered phase in the standard Vicsek model. Long-range correlations in a system governed by local interactions (such as the Ising model or the Vicsek model with boundaries) occur only close to the critical point. These long-range correlations are not enough to keep the flock together when the boundaries are removed. Furthermore, long-range correlations developed through short-range interactions do not exist in the ordered phase. Instead, as we have seen, one has to explicitly assume the existence of long-range interactions between the particles in order to prevent the flock from quickly disintegrating when it moves in open space. The important point is that just a small fraction of long-range interactions is enough for the system to reach highly ordered states and remain cohesive for long periods of time. However, from Fig. 2(b) it can be seen that the system slowly expands throughout time. To characterize the system’s expansion we use the average distance between particles Δ*d*(*t*) and the average nearest neighbor distance Δ*d*_*nn*_(*t*) defined as

4 $${\langle {\rm{\Delta }}d(t)\rangle }_{\kappa }=\langle \frac{2}{N(N-1)}\sum _{i < j}{d}_{ij}(t){\rangle }_{\kappa },$$

5 $${\langle {\rm{\Delta }}{d}_{nn}(t)\rangle }_{\kappa }=\langle \frac{1}{N}\sum _{i,j\ne i}^{N}min\{{d}_{ij}(t)\}{\rangle }_{\kappa },$$

where *d*_*ij*_(*t*) = |**r**_*i*_(*t*) − **r**_*j*_(*t*)| is the distance between the particles *p*_*i*_ and *p*_*j*_ at time *t*. Figure 4 shows that these two quantities display diffusive behavior for long times, as they asymptotically behave as *t*^*α*^ with *α* ≈ 1/2 (panels a, b, d and e in Fig. 4). Therefore, long-range interactions do not completely prevent the expansion of the system, but are able to significantly slow it down. To show that this is indeed the case, we report in Fig. 4(c,f) the diffusion coefficients associated to 〈Δ*d*(*t*)〉_*κ*_ and 〈Δ*d*_*nn*_(*t*)〉_*κ*_. The diffusion coefficient is defined as $D=\mathop{\mathrm{lim}}\limits_{t\to \infty }{\langle {t}^{-\alpha }{\rm{\Delta }}d(t)\rangle }_{\kappa }$ (similarly for 〈Δ*d*_*nn*_(*t*)〉_*κ*_). It is apparent from Fig. 4 that the diffusion coefficient can decrease several orders of magnitude by increasing *κ*, allowing the group to remain cohesive for long periods of time.

## Discussion and Concluding Remarks

Traditionally, flocking models have incorporated two main characteristics: (i) short-range alignment interactions between neighboring particles, and (ii) periodic (or confining) boundary conditions. For several years, one of the main problems investigated using these models was the emergence of states of collective order driven either by particle density or by noise. Little attention was paid to the cohesiveness of the flock. However, it has been recognized that when the periodic boundaries are eliminated, letting the particles move in open space, many models based on purely local interactions are not able to organize into a coherently moving group since even small amounts of noise destroy the flock’s order and cohesion. This happens even for initial flock densities and noise intensities that would correspond to the ordered phase in the equivalent model with periodic boundary conditions^38^. Therefore, some strategies have been put forward to prevent the flock from breaking apart. Among those strategies, the following stand out: (a) Introduce infinite-range attractive forces between all the particles^16,27^ (b) Let each particle be aware of the motion of the entire flock and move accordingly^12^. (c) Introduce a velocity bias where each particle moves in a specific way that depends on its position in the flock (i.e., whether in the bulk or on the border)^32–34^. All these strategies introduce, implicitly or explicitly, long-range interactions between the particles. Nonetheless, often in these models the long-range interactions are masked by the complicated (although perhaps realistic) forces implemented between the particles. Consequently, it is difficult to determine the role that long-range interactions have on the cohesion of the flock. Here we have investigated this role by explicitly implementing long-range alignment interactions between particles and quantifying its effect on the flock’s cohesion. It is important to emphasize that we have not introduced any type of attractive forces between the particles, but only alignment interactions, both short and long range.

The results presented here show that when just a small fraction of long-range alignment interactions per particle are introduced, the flock is able to organize into a spatially localized group and reach highly ordered states. These long-range interactions do not completely prevent the system from breaking apart, but slow down its expansion through decreasing several orders of magnitude the diffusion coefficient of the distance between neighboring particles. This allows the flock to remain ordered for quite long periods of time and be able to perform noise driven collective turns.

We have considered both metric and topological short-range interactions because there is evidence that these two types of interaction occur in nature. Insects such as cannibal crickets and locusts, interact through body-to-body contact forces^3,7,25^, whereas starlings interact topologically with the first seven (or so) neighbors^4^. In the models considered here, the expansion of the system leads to different asymptotical behaviors depending on whether the short-range interactions are metric or topological. On the one hand, for the metric case the long-time dynamics are governed by the long-range interaction network, as the metric interactions disappear once the distances between neighboring particles become larger than the interaction range. On the other hand, for the topological case both short-range and long-range interactions persist throughout the expansion of the system, since topological interactions do not depend on the value of the relative distance between particles. In both cases the short-range neighbors are constantly modified due to their intrinsic motility, whereas the long-range interaction network is constantly “rewired” to capture the sensing of different regions of the flock by each individual. As we have seen, a small fraction of long-range alignment interactions is enough to significantly decrease the expansion of the flock, whether the local interactions are metric or topological. This implies that not all particles need to receive information about the motion of the group (as in Pearce *et al*.^12^). Instead, a small fraction of birds paying attention to the motion of the entire flock suffice to keep it cohesive and ordered for a long time, which suggests that this can be a mechanism that groups may use to enhance the collective states they can develop.

We would like to emphasize that the observed effects long-range interactions have on delaying the dispersal of the flock in open space seem to be model independent. To test this, we also extended the *Inertial Spin Model*^11^ by including long-range interactions in its dynamics in exactly the same way we have done here for the standard Vicsek model (see the Supplementary Note V for the details of the implementation and the results). Although the inertial spin model also consists of topological local alignment interactions, it uses very different dynamical rules than the ones we have used here. Nonetheless, by introducing long-range interactions between the particles we were able to obtain ordered states in the inertial spin model when the flock moves in open space. Interestingly, the onset of ordered motion in this model occurred at even lower values of the average long-range connectivity *κ*, and the reduction of the diffusion coefficient of the distance between neighboring particles was also larger than the one observed in the Vicsek-like model we analyze here.

Finally, it is important to mention that long-range interactions have no place in many systems of self-propelled particles which, by their very nature, are based only on short-range interactions. This is the case for instance of the so-called soft-active matter, where self-propelled particles representing molecules interact only through short-range forces^47,48^. It has been shown that these molecules assemble faster and form larger aggregates than molecules without self-propulsion^49–51^. Likewise, self-propelled particles interacting through very simple local rules can give rise to structures resembling organic forms, such as dividing cells^52^. Self-propulsion can also speed up chemical reactions and yield interesting forms when the particles are considered as chemical reactants^53,54^. Therefore, long-range interactions are not always a desirable characteristic in groups of self-propelled particles. However, they can provide a useful strategy when cohesion of a large group is important.

## Electronic supplementary material

Supplementary Material

## Data Availability

The source code of the numerical simulations and the data supporting the findings of this study are available from the author M.Z. upon request.
