# Supplementary material for: Delay in the dispersal of flocks moving in unbounded space using long-range interactions
Source: Sci Rep. 2018 Oct 26;8:15872. doi: 10.1038/s41598-018-34208-x (PMC6203710; doi:10.1038/s41598-018-34208-x)
Supplement: Supplementary file 1 — Supplementary Material [file 41598_2018_34208_MOESM1_ESM.pdf]

Supplemental Material: Delay in the dispersal of flocks moving in unbounded space using long-range interactions

Martín Zumaya,[1,2] Hernán Larralde,[3] and Maximino Aldana[3,2,*]

[1]*Instituto de Ciencias Físicas, Universidad Nacional Autónoma de México, Avenida Universidad s/n, Colonia Chamilpa, Código Postal 62210, Cuernavaca, Morelos, México*
[2]*Centro de Ciencias de la Complejidad, Universidad Nacional Autónoma de México, Ciudad de México, México*
[3]*Instituto de Ciencias Físicas, Universidad Nacional Autónoma de México, Apartado Postal 48-3, Código Postal 62251, Cuernavaca, Morelos, México*

I. THE BEHAVIOURAL RULES MODEL OF COLLECTIVE MOTION IN OPEN SPACE

The Behavioural Rules Model introduced by Couzin et al. in [1], is a collective motion model based on metric interactions between particles, able to display coherent motion in open three dimensional space. The model consists of N individuals $\{p_1, p_2, ..., p_N\}$, each characterized by its position $\mathbf{c}_i$, and velocity $\mathbf{v}_i$ with constant speed $v = |\mathbf{v}_i|$. At every time step each particle p_i updates its direction of motion according to the orientation and position of all other particles within three non-overlapping behavioural zones around p_i. These zones, depicted in Fig.S1, are: (A) the repulsion zone (*zor*), defined as a spherical neighborhood of radius r_r centered at p_i where particles repel each other to keep a minimum distance between them. (B) The orientation zone (*zoo*), defined as a spherical shell of width $\Delta r_o = r_o - r_r$, where p_i aligns its direction of motion with the average orientation of the particles within this zone. (C) The attraction zone (*zoa*), defined as a spherical shell of width $\Delta r_a = r_a - r_o$, where p_i adjusts its direction of motion to point towards the average position of the particles in this zone.

Let us denote as $\mathbf{d}_i(t)$ the direction vector of particle p_i at time t, and define the repulsion, orientation and attraction vectors associated to this particle, respectively, as

$$\mathbf{d}_i^r(t+\Delta t) = -\sum_{j\neq i}^{n_i^r(t)} \frac{\mathbf{r}_{ij}(t)}{|\mathbf{r}_{ij}(t)|}, \tag{S1a}$$

$$\mathbf{d}_i^o(t+\Delta t) = \sum_{j=1}^{n_i^o(t)} \frac{\mathbf{v}_{ij}(t)}{|\mathbf{v}_{ij}(t)|}, \tag{S1b}$$

$$\mathbf{d}_i^a(t+\Delta t) = \sum_{j\neq i}^{n_i^a(t)} \frac{\mathbf{r}_{ij}(t)}{|\mathbf{r}_{ij}(t)|} \tag{S1c}$$

where $n_i^r(t), n_i^o(t)$ and $n_i^a(t)$ are the number of neighbors in each behavioral zone at time t, $\mathbf{r}_{ij} = (\mathbf{c}_i - \mathbf{c}_j)/|\mathbf{c}_i - \mathbf{c}_j|$ is the unit vector from p_i to its neighbor p_j, and Δt is the integration step. These vector determine the interaction of particle p_i with all the particles within the corresponding behavioural zones.

* max@icf.unam.mx

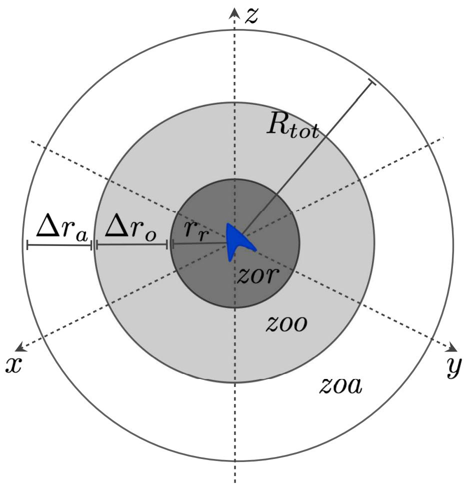

FIG. S1: Behavioural Rules Model. The interaction between particles depends on which zone they are located. In the repulsion zone (zor) particles repel each other in order to keep a minimum distance between them. In the orientation zone (zoo), particles align their direction of motion. In the attraction zone (zoa) particles move towards the average position of all the other particles in this zone.

The direction of p_i is then updated according to the rules

$$\mathbf{d}_i(t+\Delta t) = \mathbf{d}_i^r(t+\Delta t) \text{ if } n_r > 0, \tag{S2a}$$

$$\mathbf{d}_i(t+\Delta t) = \mathbf{d}_i^o(t+\Delta t) \text{ if } n_r, n_a = 0, \tag{S2b}$$

$$\mathbf{d}_i(t+\Delta t) = \mathbf{d}_i^a(t+\Delta t) \text{ if } n_r, n_o = 0, \tag{S2c}$$

$$\mathbf{d}_i(t+\Delta t) = \frac{1}{2}[\mathbf{d}_i^o(t+\tau) + \mathbf{d}_i^a(t+\tau)] \quad \text{if } n_r = 0 \text{ and } n_o, n_a \neq 0. \tag{S2d}$$

In the case that all the interactions add up to zero or no other particles are detected within the behavioural zones, the particle does not modify its direction of motion, i.e.

$$\mathbf{d}_i(t+\Delta t) = \mathbf{v}_i(t). \tag{S3}$$

Finally, to bring the Behavioural Rules Model and the one we propose on a common ground, the introduction of the noise in the system and the update of the positions and velocities of all particles is done in the same manner as is described in Eq. 2 of the main text. The Behavioural Rules Model also considers a maximum turning rate $\theta\Delta t$ which defines the maximum angle each particle can modify its direction of motion at every

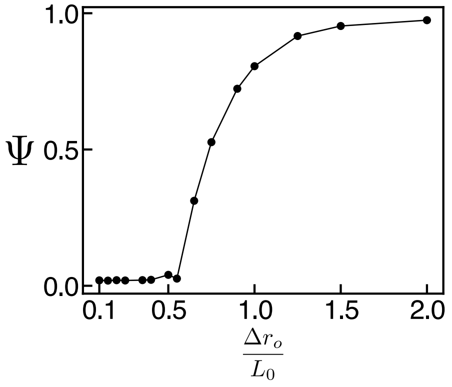

FIG. S2: Order parameter Ψ for the Behavioural Rules Model in open space as a function of the orientation range Δr_o, for a system with $N = 2000$ and initial size $L_0 = 19$. The repulsion and attraction ranges r_r and Δr_a were kept fixed at the values $r_r = 1$ and $\Delta r_a = L_0 = 19$. It is apparent from this figure that only when $\Delta r_o \approx \frac{3}{2} L_0$ the order parameter Ψ reaches values corresponding to ordered states: $\Psi \approx 1$. The value of the other parameters used to generate this data are listed in Table S1.

Behavioural Rules Model		
N	Number of particles	2000
L_0	Initial size of the flock	19
r_r	Radius of the repulsion zone	$1.0 \approx 0.05 L_0$
$\Delta r_o / L_0$	Size of the orientation zone relative to system size	$[0.1, 2.0]$
$\Delta r_a / L_0$	Size of the attraction zone relative to system size	1.0
η	Noise Intensity	0.15
ρ_0	Initial density	0.3
v_0	Speed of the particles	1.0
θ	Maximum turning rate	360°
t_∞	Maximum computing time	10^5

TABLE S1: Parameters used in the numerical simulations of the Behavioural Rules Model.

Standard Vicsek Model (2D and 3D)		
N	Number of particles	4096
η	Noise intensity	0.15
v_0	Particle speed	1.0
Δt	Integration step	1.0
t_∞	Maximum time to compute trajectories	10^6
r_0	Metric interaction range	$2 v_0 \Delta t$
ρ_0	Initial density	0.3
L_0	Initial size of the box	$\sqrt[d]{N/\rho_0}$

TABLE S2: Parameters used in the numerical simulations of the SVM.The initial density ρ_0 and initial box size L_0 were used for the SVM in open space (without boundaries). In this case, N particles with random orientations were initially placed in a box of size L_0. After that, the particles moved without boundaries. In the definition of L_0, d is the dimensionality of the space.

time step. We performed numerical simulations in open space for the Couzin model described above using different values of Δr_o while keeping Δr_a, r_r and the noise intensity η fixed, and starting from random initial conditions. The sizes of the different behavioural zones were defined relative to the initial system size L_0, which is defined by the initial density ρ_0 by $L_0 = \sqrt[3]{N/\rho_0}$. The parameters used in the numerical simulations are listed in Table S1. As depicted in Fig.S2, for the system to be able to reach ordered states both the *zoo* and *zoa* must be each comparable to the system size L_0. Moreover, the total interaction region ($R_{tot} = r_r + \Delta r_o + \Delta r_a$) for each particle must be considerably larger than L_0. This leads to the situation where essentially all particles in the system interact with all other ones, which is in clear contrast with the model we propose here, where just a few long-range interactions are needed to reach ordered and cohesive states in open space.

II. PARAMETER VALUES

Tables S2 and S3 summarize the values of the parameters used in the numerical simulations of the different models described in the text (unless otherwise stated). In what follows we will refer to the standard Vicsek model as SVM, and to the flocking model with short-range and long-range interactions moving in open space as FMSLRI.

III. NOISE INDUCED ORDER-DISORDER TRANSITION IN THE FMSRLI

The objective of this work is to characterize the effect of long-range interactions on the separation of the particles due to their motion in open space. However, it is important to mention that a noise-driven order-disorder phase transition also exists in this case for both metric and topological short-range interactions, as Fig. S3 shows. Therefore, introducing long-range interactions does not only keep the flock together in open space, but allows the system to undergo the familiar order-disorder transition observed in the Viscek model with boundaries, which otherwise would not exist. In the numerical simulations presented in the text, we used the constant value $\eta = 0.15$ for the noise intensity, which in all cases corresponds to the ordered phase (see Fig. S3).

Flocking model with short-range and long-range interactions in open space (2D and 3D)

N	Number of particles	4096
η	Noise intensity	0.15
v_0	Particle speed	1.0
Δt	Integration step	1.0
t_∞	Maximum time to compute trajectories	10^6
ω	Relative weight between short-range and long-range interactions	0.5
α_l	Number of first topological neighbors	6
r_0	Metric interaction range	$2v_0\Delta t$
ρ_0	Initial density	0.3

TABLE S3: Parameters used in the numerical simulations of the FMSLRI.

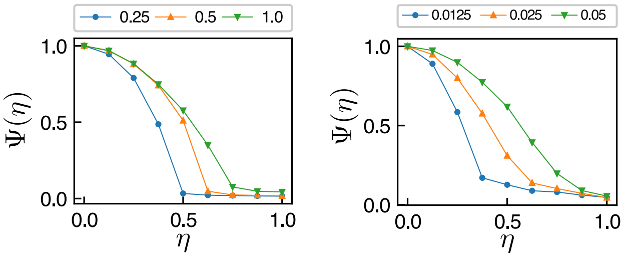

(a) (b)

FIG. S3: Order-disorder transition in the FMSRLI driven by the noise intensity η for systems with $N = 4096$ particles and fixed initial density $\rho_0 = 0.3$. (a) Metric short-range interactions and long-range connectivity $\kappa = 0.25, 0.5, 1.0$. (b) Topological short-range interactions and long-range connectivity $\kappa = 0.0125, 0.025, 0.05$.

IV. DYNAMICS OF THE FLOCKING MODEL IN TWO DIMENSIONS.

In the main text we presented results in 3D. However, numerical simulations were also performed in 2D. The short-range interactions were implemented both metrically and topologically. As for the 3D case, we chose values of the initial density ρ_0 and noise intensity η that correspond to the ordered phase of the standard Vicsek model in 2D with periodic boundary conditions.

In two dimensions, the model consists of N self-propelled particles $\{p_1, p_2, \ldots, p_N\}$ characterized by its position $\mathbf{r}_n$ and velocity $\mathbf{v}_n = v_0 e^{i\theta_n(t)}$, expressed as a complex number to emphasize its direction $\theta_n(t)$, and the speed of all the particles, $||\mathbf{v}_n(t)|| = v_0$ is kept cosntant at all times.

The evolution of the system is given by the simultaneous update of the directions and positions of all the particles in the system according to the rules

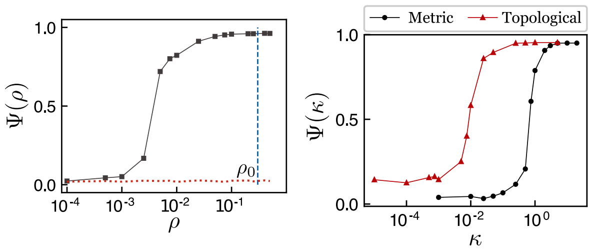

(a) (b)

FIG. S4: Order-disorder phase transition for systems with $N = 4096$ particles, fixed noise intensity $\eta = 0.15$ and initial density $\rho_0 = 0.3$. (a) Ψ as a function of the density ρ for the 2D SVM. (b) Ψ as a function of the long-range connectivity κ for the 2D FMSLRI. Metric and topological short-range interactions correspond to circles and triangles, respectively.

$$\theta_i(t+\Delta t) = \omega \operatorname{ang}[\mathbf{V}_i(t)] + (1-\omega) \operatorname{ang}[\boldsymbol{\Upsilon}_i(t)] + \eta\, \xi_i(t), \quad \text{(S4a)}$$

$$\mathbf{v}_i(t+\Delta t) = v_0\, e^{i\theta_i(t+\Delta t)}, \quad \text{(S4b)}$$

$$\mathbf{r}_i(t+\Delta t) = \mathbf{r}_i(t) + \mathbf{v}_i(t+\Delta t)\,\Delta t, \quad \text{(S4c)}$$

where the function ang$[\mathbf{u}] = \theta$ for every vector $\mathbf{u} = u_0 e^{i\theta}$. The remaining parameters ω, η and the variable $\xi_i(t)$ have the same meaning as in the 3D case, and the results presented corrsespond to $\omega = 1/2$.

Fig.S4a shows the order parameter Ψ as a function of the density ρ for the SVM in 2D with periodic boundary conditions (black curve) and in open space (red dotted line). Again, it can be seen that when the flock is moving in open space, it cannot reach ordered states for any value of the particle density. Fig.S4b shows Ψ as a function of the average long-range connectivity κ for the FMSLRI when the short-range interactions are implemented metrically (black curve with circles) and topologically (red curve with triangles). In both cases the system is able to reach ordered states even for relatively small values of κ.

Examples of particles' trajectories for different values of the mean long-range connectivity can be seen in Fig.S5a, Fig.S5b and Fig.S5c for metric short-range interactions, and in Fig.S6a, Fig.S6b and Fig.S6c for topological short-range interactions. In both cases the trajectories show that the particles are organized in an ordered spatially localized group, able to perform noise induced collective changes of direction.

As it is depicted in Fig.S4b, there is an order-disorder transition as a function of the mean long-range connectivity κ. It is to be noted that the system is not able to reach an ordered state in the absence of long-range interactions, even for densities in the ordered phase of the Vicsek Model, and that very few long-range interactions per particle are needed to achieve and ordered state in unbounded collective motion.

The figures in Fig. S5 and Fig. S6 show typical trajectories and characterize the expansion of the FMSLRI in 2D open space with respect to the average long-range connectivity κ. The behavior is completely analogous to the one presented

in the main text, which corresponds to 3D open space. This shows that long-range connections between just a small fraction of particles suffice to generate ordered states and keep the flock together for long periods of time, either in 2D or 3D and with metric or topologic short-range interactions.

V. EXTENDED INERTIAL SPIN MODEL

To further test the generality of our results, we extended the *inertial spin model* introduced in [2] by implementing long-range interactions in a completely analogous way as we did for the FMSLRI. The inertial spin model is also based on topologic short-range interactions between the particles, but the interaction rules are very different from the ones implemented in the Vicsek model (or the FMSLRI).

The inertial spin model, as it is presented in [2], consists of N particles $\{p_1, p_2, \ldots, p_N\}$, characterized by its position $\mathbf{r}_i$, velocity $\mathbf{v}_i$ (with the same speed $\|\mathbf{v}_i\| = v_0$ for all particles), and a generalized momentum variable $\mathbf{s}_i$, referred to as the spin of the particle. This last variable is related to the instantaneous curvature of the particle's trajectory and serves as the intermediary of the interactions between particles through which they modify their direction of motion. Each particle p_i interacts with its first α_l neighbors (topologic short-range interactions), which constitute its local neighborhood $U_i(t)$. In order to extend this model and include long-range interactions, we randomly choose κ_i particles out of the $N - \alpha_l$ particles remaining outside the local neighborhood $U_i(t)$, where κ_i is a random number drawn from a Poisson distribution with average κ. This set of κ_i particles constitute the long-range neighborhood $L_i(t)$ of particle p_i. Assigning a long-range neighborhood to each particle in the system generates a random network of long-range interactions with average degree κ.

Once each particle has been provided with a local neighborhood $U_i(t)$ and a long-range neighborhood $L_i(t)$, the temporal evolution of the system is given by the simultaneous update of the positions and velocities of all the particles according to the rules

$$\frac{d\mathbf{v}_i(t)}{dt} = \frac{1}{\chi}\mathbf{s}_i(t) \times \mathbf{v}_i(t) \quad \text{(S5a)}$$

$$\frac{d\mathbf{s}_i(t)}{dt} = \mathbf{v}_i(t) \times \Big\{ \frac{J}{v_0^2}\Big[\mathbf{V}_i(t) + \boldsymbol{\Upsilon}_i(t)\Big] - \frac{\eta}{v_0^2}\frac{d\mathbf{v}_i}{dt} + \frac{\boldsymbol{\xi}_i}{v_0}\Big\} \quad \text{(S5b)}$$

$$\frac{d\mathbf{r}_i(t)}{dt} = \mathbf{v}_i(t), \quad \text{(S5c)}$$

where $\mathbf{V}_i$ and $\boldsymbol{\Upsilon}_i$ are respectively the short-range and long-range signals defined in Eq.1 of the main text. The parameter χ is a generalized momentum of inertia, η represents a friction coefficient, and J is the strength of the alignment force between neighbors. The random vector $\boldsymbol{\xi}$ represents noise in the system with correlation

$$\langle \boldsymbol{\xi}_i(t) \cdot \boldsymbol{\xi}_k(t) \rangle = (2d)\, \eta\, T \delta_{ij} \delta(t - t') \quad \text{(S6)}$$

where T plays the role of a generalized temperature. From Eq.S5 it can be seen that we are introducing short and long-range interactions at equal footing. This does not need to be the case and, as in the FMSLRI, a weight factor ω between short and long-range interactions can be introduced (as in Eq.2 of the main text), which would give more relevance to one type of interaction versus the other. Nonetheless, for simplicity we have considered again $\omega = 1/2$.

It is not our objective here to give a comprehensive description of the parameters of the inertial spin model as they can be consulted in [2]. However, it is important to mention that the system can operate in two different dynamical regimes depending on the values of the parameters. The first one is the *overdamped regime*, in which the information each particle receives about the orientation of the other particles decays exponentially with distance. In this case, perturbations in the orientation of the particles do not propagate across the system, preventing the system from performing collective turns. The other dynamical regime is the *underdamped regime*, in which the information about the orientation of the particles decays linearly with distance. In this case perturbations in the orientation of the particles can propagate across the system, producing collective turns. Here we present numerical simulations of the extended inertial spin model (i.e. the model in which long-range interactions have been introduced) in the underdamped regime. The values of the parameters we used are listed in Table S4.

Extended Inertial Spin Model		
N	Number of particles	512
χ	Generalized momentum of inertia	1.25
η	Friction coefficient	1.5
J	Strength of the alignment force	0.8
T	Generalized Temperature	0.01
κ_l	Number of first topological neighbors	6
ρ_0	Initial density	0.3
v_0	Speed of the particles	0.1
Δt	Integration step	$v_0\sqrt{J/\chi}$
t_∞	Maximum computing time	10^6

TABLE S4: Parameters used in the numerical simulations of the extended inertial spin model.

Figs. S7a, b and c show typical trajectories of the extended internal spin model (EISM) starting from random initial conditions in open space for different values of the average long-range connectivity κ. Again, when $\kappa = 0$, i.e. in the absence of long-range interactions, the system is able neither to reach ordered states nor to remain together (Fig.S7a). However, even for very value as small as $\kappa = 0.005$ the flock moving in open space can develop ordered states and remain together for long periods of time (Fig.S7b). Increasing the value of κ only makes the system more stable (Fig.S7c).

Fig. S7d shows the order parameter Ψ as a function of κ for the EISM moving in open space. This is the familiar phase-transition curve driven by the average number of long-range interactions per particle. Remarkably, the onset of ordered states in this model begins for very low values of κ, about one order of magnitud lower than for the FMSLRI discussed in the main text.

Figs. S7e and f show the average distance between particles $\langle \Delta d \rangle_\kappa$ and to the first neighbor $\langle \Delta d_{\mathbf{nn}} \rangle_\kappa$ as functions of time for increasing values of κ. Although asymptotically these quantities behave as $t^{1/2}$, the diffusion coefficient rapidly decreases several orders of magnitude as κ increases. Note that for relatively large values of κ the curves for $\langle \Delta d \rangle_\kappa$ and $\langle \Delta d_{\mathbf{nn}} \rangle_\kappa$ remain flat during most of the computing time $t_\infty = 10^5$.

We want to stress that the results obtained for the EISM correspond to random initial conditions, i.e. both the positions and orientations of the particles were assigned randomly at the beginning of the simulation. This is in contrast to the simulations reported in [2] where the system was initialized in an already ordered and localized state. Also, the noise intensity we used here was considerably higher than the one used in [2].

Our results show the ability of long-range interactions to build up ordered states and delay the fracture of the system for long times in unbounded space. Moreover, we have shown that these features are not specific of a particular model, but hold for different models such as the Vicsek model with metric and topological short-range interactions and the inertial spin model (which implements topological short-range interactions). In all these cases the system cannot reach ordered states in open space without long-range interactions between some particles. Interestingly, in all the models presented here, the fraction of particles with long-range interactions necessary to reach develop ordered states and keep the flock together is very small: only one long-range connection per 100 (or even 1000) particles.

[1] I. D. Couzin, J. Krause, R. James, G. D. Ruxton, and N. R. Franks, Journal of Theoretical Biology **218**, 1 (2002).

[2] A. Cavagna, L. D. Castello, I. Giardina, T. Grigera, A. Jelic, S. Melillo, T. Mora, L. Parisi, E. Silvestri, M. Viale, and A. M. Walczak, J. Stat. Phys. **158**, 601 (2014).

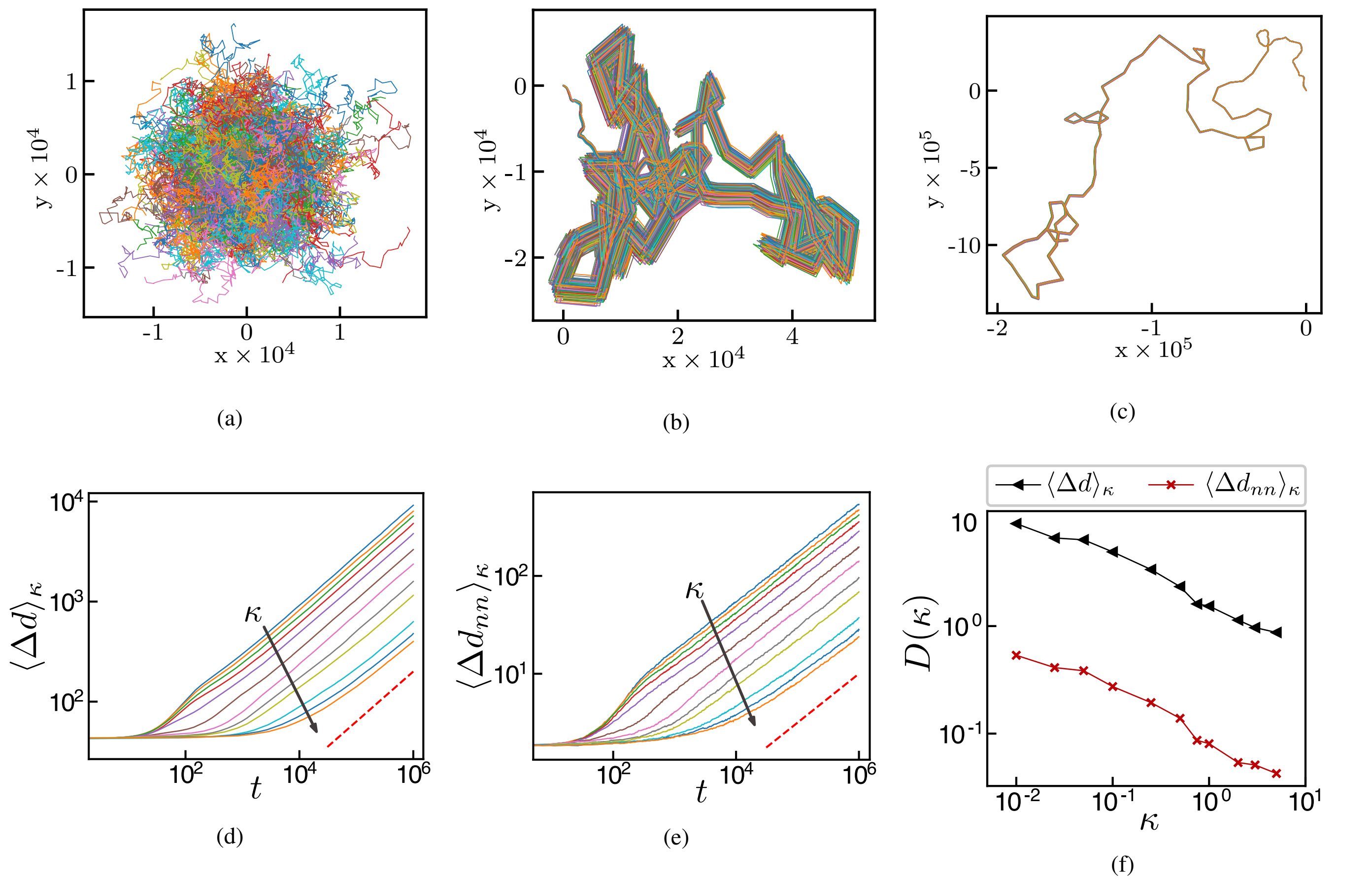

FIG. S5: Typical trajectories in 2D open space of the FMSLRI with $N = 512$ particles and different values of the average long-range connectivity: (a) $\kappa = 0$, (b) $\kappa = 1$ and (c) $\kappa = 5$. In this figure the short-range interactions were implemented metrically. Panels (d) and (e) show the average distance between particles $\langle \Delta d(t) \rangle_\kappa$ and between nearest neighbors $\langle \Delta d_{nn}(t) \rangle_\kappa$ as functions of time for different values of κ. The arrow indicates the direction of increasing κ in the interval $[0.001, 20.0]$. Note that these distances behave asymptotically as $t^{1/2}$ (red dashed line), indicating diffusive behavior. (f) Diffusion coefficients for $\langle \Delta d(t) \rangle_\kappa$ and $\langle \Delta d_{nn}(t) \rangle_\kappa$ as functions of κ. Note the decrease in about one order of magnitude as κ varies from $\kappa = 0.01$ up to $\kappa = 1$. The values of the other parameters used in the simulation are listed in Table S3.

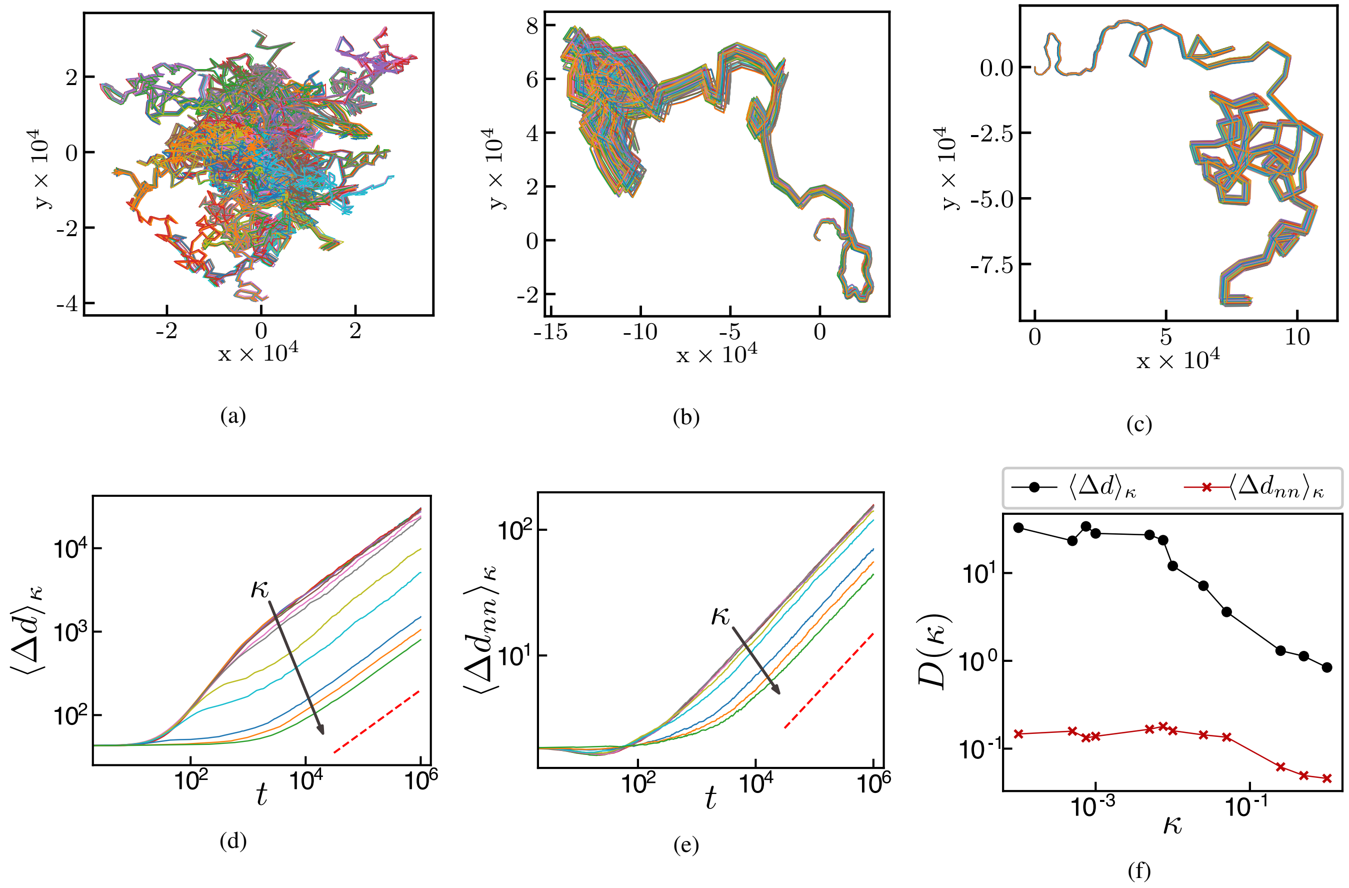

FIG. S6: Same type of plots as in Fig. S5 for the FMSLRI in 2D open space, but this time the short-range interactions were implemented topologically (a) $\kappa = 0$, (b) $\kappa = 0.05$, (c) $\kappa = 0.25$. The arrow indicates the direction of increasing κ in the interval $[10^{-5}, 1.0]$.

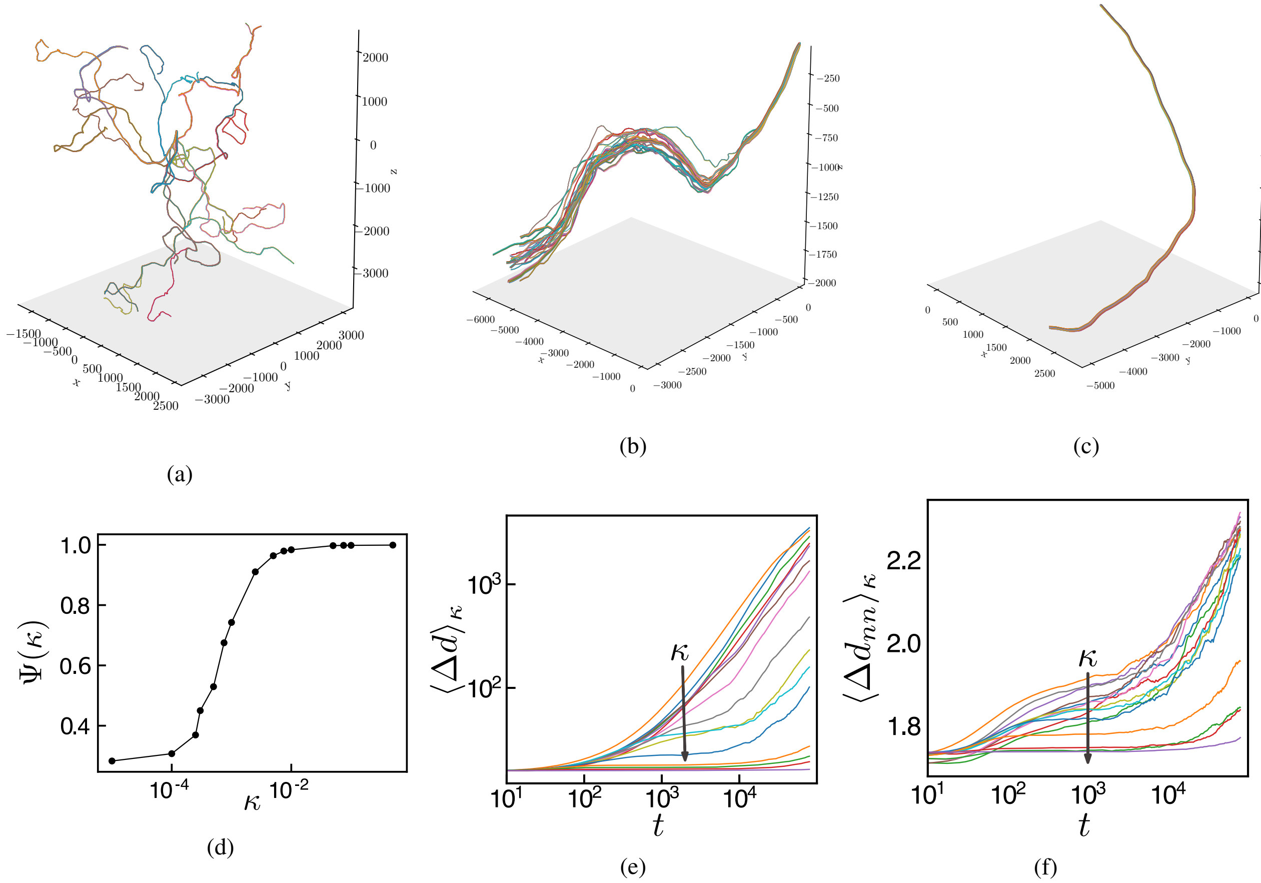

FIG. S7: Typical trajectories of the underdamped Extended Internal Spin Model (EISM) moving in open space for different values of κ: (a) $\kappa = 0$, (b) $\kappa = 0.005$ and (c) $\kappa = 0.075$. The number of particles in these simulations was $N = 512$. Values for the other parameters are listed in Table S4. (d) Order-disorder transition driven by the average long-range connectivity κ. Note the onset of ordered states for values of κ as low as $\kappa = 0.001$. Panels (e) and (f) show the temporal evolution of the average distances $\langle \Delta d(t) \rangle_\kappa$ and $\langle \Delta d_{nn}(t) \rangle_\kappa$ (the arrows indicate the direction of increasing κ in the interval $[0.0, 0.5]$). Note that as κ increases these curves become flatter and flatter, indicating an almost zero diffusion coefficient.
